# Specific Antibody and Interferon-Gamma Responses Associated with Immunopathological Forms of Bovine Paratuberculosis in Slaughtered Friesian Cattle

**DOI:** 10.1371/journal.pone.0064568

**Published:** 2013-05-28

**Authors:** Patricia Vazquez, Joseba M. Garrido, Ramon A. Juste

**Affiliations:** Department of Animal Health, NEIKER-Tecnalia, Derio, Bizkaia, Spain; University College Dublin, Ireland

## Abstract

*Mycobacterium avium* subsp. *paratuberculosis* (MAP) infection causes a chronic granulomatous inflammatory regional enteritis in ruminants. Cell-mediated immune responses are assumed to be protective and therefore, to be associated with its more delimited lesion types, while humoral responses are mainly associated with *diffuse* histopathological lesions. However, this duality of immune responses has been recently questioned. The aim of this study was to assess the relationship between both types of immunological responses and the type and extension of intestinal lesions and the presence of MAP in bovine tissues. Standard histopathological examinations, two microbiological procedures (culture and real time PCR (rtPCR)), as well as MAP specific antibody and interferon gamma (IFN-γ) release assays (IGRA) were performed on tissues and blood of 333 slaughtered Holstein-Friesian animals. Paratuberculous lesions were observed in 176 (52.9%) of the animals and overall MAP detection rates were estimated at 13.5% and 28.5% for tissue culture and rtPCR, respectively. Unlike the relatively constant non-specific IFN-γ release, both the antibody levels and the specific IFN-γ release significantly increased with tissue damage. Delimited immunopathological forms, which accounted for 93.2% of all forms, were mostly related to positive testing in the IGRA (38.4%) whereas *diffuse* ones (6.8%) were associated with antibody seropositivity (91.7%). However, since the frequency of positive immune responses in both tests increased as the lesions severity increased, polarization of Th1/Th2 responses was less prominent than expected. MAP was detected in the majority of ELISA-positive animals (culture+: 90%, rtPCR+: 85%) but the bacteria was only confirmed in the 36.1% of IGRA-positive animals by any of the two microbiological tests. In terms of diagnosis, the antibody test was a good indicator of advanced tissue damage (*diffuse* forms), but the IGRA did not associate well with more delimited forms or with MAP detection.

## Introduction

Infection with *Mycobacterium avium* subsp. *paratuberculosis* (MAP) leads to a slow and progressive granulomatous enteritis and lymphadenitis, known as Paratuberculosis (PTB) or Johne’s disease, particularly affecting domestic and wild ruminant species [1].

In dairy cattle, MAP infected cows that present typical clinical symptoms, that is, diarrhea, poor body condition and decreased milk production, are mainly those in their first and second calving. However, most infected animals would remain as unapparent MAP carriers because they do not develop clinical disease, and microbiological and immunological diagnostic tests are not sensitive enough to identify them [2]. This might be a consequence of a natural resistance against MAP where infection would be restricted to *focal* forms without clinical disease [3].

The immunological events occurring in ruminant PTB have been studied regarding the development of inflammatory lesions in the small intestine and associated lymph-nodes (LN) and the progression to clinical disease and MAP shedding [4]–[7]. In the initial stages of infection, MAP usually triggers a predominantly pro-inflammatory and cytotoxic cytokine pattern so as to contain the progress of infection [8]. This Th1 cell-mediated response is mainly characterized by the release of interferon-gamma (IFN-γ), interleukin-2 (IL-2) and tumor necrosis factor-alpha (TNF-α) [9]–[10]. In fact, IFN-γ has a relevant role in determining both a correct Th1-cell differentiation and macrophage activation [11]. These pathways, as well as the onset of adequate innate and adaptive immune responses, like those seen in human inflammatory bowel disease (IBD) and other mycobacterial diseases, appear to be associated with a genetic component. There is overwhelming evidence suggesting that resistance to bovine PTB may be conferred by certain polymorphisms of immunity related genes and pattern recognition receptors (PRRs) [12], which has been also supported by a recent meta-analysis of two genome-wide association studies of two cohorts of Holstein cows (USA and Italy) [13]. However, if MAP reactivation occurs or the host immune system is weakened, these proposed resistant forms (*focal* lesions) can shift to *diffuse* forms and clinical disease of fatal consequences [14]. In these cases, the Th1-type response is overcome by a non-protective IgG1 mediated response (Th2–type) and the IFN-γ levels are reduced mainly because of the effect of two anti-inflammatory cytokines: the interleukin-10 (IL-10) and the transforming growth factor beta (TGF-β) [8]. Recent work in ovine PTB, has pointed out that this model might not be so simple and that the immunopathology of PTB needs to be reviewed [15].

Although the antibody-based response is still not fully elucidated for most MAP specific antigens [16], it is well-known that humoral responses are associated with clinical manifestations and large amounts of bacterial shedding [17], [18]. For this reason, nowadays the use of the ELISA test remains helpful for PTB control because of its good performance for infectious animal detection at a low cost [19]. The IFN-γ release assay (IGRA) has been long time considered to be a promising tool for identifying early stages for MAP infections but without never totally fulfilling these expectations. Although intensive efforts have been made to improve the sensitivity of the test and good prospects have been reported for assessing MAP exposure rate of cattle younger than 1 year of age [20], its predictive diagnostic value is still rather questionable because infected adult cattle appear to develop fluctuant cell-mediated responses, as a consequence of efficient elimination of mycobacteria or on the contrary, of disease progress [21]–[23]. Additionally, exposure events to other *Mycobacterium* spp. might lower the test specificity. Then, repeated testing or complementary methods (e.g. fecal culture or PCR) are required to achieve reasonable sensitivity for PTB diagnosis.

In the present study, the patterns of association between immunological responses, MAP detection in tissues and pathological findings in adult cattle naturally infected with MAP are studied and discussed from both diagnostic and epidemiologic perspectives.

## Methods

### Ethics Statement

Animals used in this study were not submitted to any *in vivo* manipulation prior to stunning for industrial slaughter and therefore, no specific ethics committee authorization was required.

### Animals and Sampling

A total of 333 slaughtered Friesian cattle older than 24 months of age (on average 4.5 years-old) were included in this study (Table 1). These animals belonged to herds located in North-East of Spain (Table 2) and were slaughtered in the Bilbao municipal slaughterhouse (Bilbao, Bizkaia, Basque Country, Spain) between February 2008 and March 2010. This slaughterhouse was operated by a municipally owned company and complied with the pertinent Basque (Basque Government Decree 454/1994), Spanish (Spanish Government Law 32/2007 and Royal decree 731/2007) and European (Council Regulation (EC) No 1099/2009) legislation on animal welfare under the supervision municipal veterinarians. The works involved in sampling as well as the use of the material obtained was properly authorized by the slaughterhouse managers.

**Table 1:** Animals: Age at slaughter.

	North-East of Spain									Other places
% Animals	Aragon	Asturias	Basque Country	Cantabria	Castilla y Leon	Catalonia	Galicia	La Rioja	Navarra	EU^1^ countries
**At birth**	0.6	0.6	25.8	15.3	6.0	43.8	0.3	0.0	0.6	6.9
**Pre-slaughter**	0.9	0.9	29.7	14.4	4.8	47.4	0.0	1.8	0.0	0.0

Age data are shown in months (yearly intervals).

**Table 2:** Animals: Origin.

	Age at slaughter (Months)								
	24.0–35.9	36.0–47.9	48.0–59.9	60.0–71.9	72.0–83.9	84.0–95.9	96.0–107.9	108.0–119.9	120.0–131.9
**% Animals**	8.1	27.3	32.7	19.8	8.7	2.4	0.0	0.6	0.3

Origins of animals are shown according to the information registered in the bovine identification system (Council Regulation (EC) No 820/97): place of birth and the last location before slaughter. EU^1^ = Belgium, France, Germany, Holland and Italy.

Sampling was systematically performed once a week at the slaughterhouse. In each visit, the first 2 to 10 animals in the line satisfying the breed and age requirements were sampled (on average 5 animals/sampling). Right after stunning and before bleeding, duplicate blood samples from the jugular vein were collected into two 10 mL tubes containing lithium heparin or EDTA (BD Vacutainer®, Franklin Lakes, NJ, USA), respectively. Additionally, small intestine from each animal was picked up and taken to NEIKER-Tecnalia necropsy room where samples from the ileum and two associated mesenteric (jejunal caudal and ileocecal) lymph nodes (LN) were taken for histopathological and microbiological investigation (ileum and jejunal LN).

### Histopathological Examination

From each animal, a sample from each LN, ileocecal valve (ICV) and contiguous distal ileum (DI) was fixed in 10% neutral-buffered formalin, and conventionally dehydrated and embedded in paraffin for cutting 4 µm thick sections. Subsequently, the sections were dehydrated and stained with haematoxylin-eosin (HE) and submitted to microscopic examination. When histopathological lesions consistent with PTB were detected, a matched section was stained with a tissue section modified Ziehl-Neelsen (ZN) procedure for acid fast bacteria (AFB).

According to location, extension and cell composition, intestinal lesions were classified as *focal, multifocal, diffuse lymphocytic, diffuse intermediate* and *diffuse multibacillary* forms, as proposed by González et al. (2005) [24].

### MAP Detection in Tissues: Culture and Real Time PCR (rtPCR)

Scraped ileal mucosa from the ICV-DI area and minced jejunal caudal LN were mixed in the same proportion and processed for isolation and PCR. Briefly, MAP isolation was performed in duplicate home-made Herrold’s egg yolk (Becton Dickinson, Franklin Lakes, NJ, USA) and Lowenstein-Jensen media (Difco, Detroit, MI, USA), both supplemented with 2 mg/L of mycobactine J (Allied Monitor, Fayette, MO, USA), as previously described [25]. A positive result was considered if one or more MAP colonies were observed in any of the four medium slants. MAP isolates were confirmed by IS*900* PCR reaction [26]. Specific MAP IS*900* DNA detection from tissues was assessed by using the combined Adiapure®-Adiavet® extraction and amplification kit (Adiagene, Saint Brieuc, France) and the ABI prism 7000 Sequence Detection System (Applied Biosystems, Forter City, CA, USA). Samples showing amplification curves with a threshold cycle (Ct) bellow 40.00 were considered positive. Details on bacteriological culture as well as MAP specific IS*900* DNA extraction and amplification protocols from tissue samples have been published elsewhere [3].

### Humoral Immune Response: ELISA Test

Serum samples were tested for specific antibodies against MAP using a two-step commercial ELISA (Pourquier® ELISA paratuberculosis antibody screening and Pourquier® ELISA paratuberculosis antibody verification. Institut Pourquier, currently IDEXX Paratuberculosis Screening Ab Test and IDEXX Paratuberculosis Verification Ab Test; IDEXX Laboratories, Inc., Westbrook, ME, USA) according the manufacturer’s instructions. This assay includes a serum *Mycobacterium phlei* pre-absorption step to correct for non-specific reactions [27]. The results were expressed as optical density (OD) values and categorized into positive and negative results, according to the transformation of OD values into sample/positive ratios (S/P), as directed by the manufacturer.

### Cell-mediated Immune (CMI) Response: IFN-γ Release Assay (IGRA)

Blood stimulation was performed within the first eight hours after blood collection. Briefly, four 1.4 mL aliquots of lithium heparinized whole blood samples from each animal were stimulated in three 24-well culture plates (Becton Dickinson, Franklin Lakes, NJ, USA) with 100 µl of phosphate-buffered saline (PBS), 100 µL of avian purified protein derivative (PPD_AV_) (0.3 µg/µL) (CZ Veterinaria® SA, Porriño, Spain) or 100 µL of bovine purified protein derivative (PPD_BOV_) (0.3 µg/µL) (CZ Veterinaria® SA, Porriño, Spain), respectively. After incubation for 16–24 h at 37°C +5–7% CO2, plasma was separated by centrifugation and frozen at −20°C until testing. Subsequently, a commercial IGRA (Bovigam™, Prionics, Schlieren, Switzerland) was performed in accordance with the manufacturer’s instructions. Results were categorized into PTB positive if the response to the PPD_AV_ was higher than the response to the PPD_BOV_ and the OD value for the PPD_AV_ after subtracting the value for the null antigen (PBS) was ≥0.05.

In turn, results were considered to be *Mycobacterium bovis* (*M. bovis*) positive if the response to the PPD_BOV_ was higher than the response to the PPD_AV_ and the OD value for the PPD_BOV_ after subtracting the value for the null antigen (PBS) was ≥0.05. This cut-off value was established as recommended by the Spanish tuberculosis (TB) control program [28], [29].

### Statistical Analysis

Statistical analyses for hypothesis testing on differences in immunological tests results as an additional support of immunopathological groups description were performed using SAS 9.1 software (SAS Institute, Cary, NC, USA). ELISA mean OD values and standard error (SE) estimations of humoral and cell-mediated immunity (CMI) responses (IGRA) were treated as quantitative dependent variables in analysis of variance according to the following model: ***ELISA/IGRA result = Immunopathological group level+error***. These variables were also categorized according to the above described specific cut-off values and treated as categorical dependent variables in frequency association tests. Immunopathological group means were compared with the LSMEANS (least square or marginal means) statement for statistical significance using the Student’s t test with the Tukey-Kramer adjustment for multiple comparisons (Proc GLM – General Linear Model SAS procedure). Fisher’s exact test (**fisher** option of the TABLES statement in the SAS Proc FREQ) was used to compare frequencies for categorical immunological variables related to pathological status (PTB lesions vs. No Lesion) and microbiology (Culture+ vs Culture-; rtPCR+ vs rtPCR-). In order to evaluate both immunological tests for “in vivo” diagnosis of immunopathological forms and MAP infection, sensitivity and specificity values were calculated taking these as the reference classification. Agreement between tests was assessed with the Kappa (κ) index (**agree** option of the TABLES statement in the SAS Proc FREQ) and interpreted as follows: 0.00–0.20 poor, 0.21–0.40 fair, 0.41–0.60 moderate, 0.61–0.80 good and 0.81–1.00 excellent. For all analyses, a p value of <0.05 was considered to be statistically significant.

## Results

### Histopathology and MAP Detection

Granulomatous inflammatory lesions consistent with MAP infection were observed in 52.9% of the studied animals (176/333) (Table 3). Most of the lesions corresponded to *focal* forms (86.4%; 152/176) while *multifocal* and *diffuse* forms accounted for 13.6% (24/176). The distribution of *diffuse* forms was as follows: 4.0% *diffuse multibacillary* (*histiocytic* infiltrate) (7/176), 2.3% *diffuse intermediate* (4/176) and 0.6% *diffuse lymphocytic* (*lymphoplasmacytic* infiltrate) (1/176). Animals without visible histopathological lesions were considered apparently free of PTB.

**Table 3:** Summary of humoral and cell-mediated responses against MAP according to histopathological findings.

		% MAP detection		ELISA		IGRA				
Histopathology	n	Culture+	rtPCR+	%Pos	AB (Mean OD±SE)	%PPD_AV_+	%PPD_BOV_+	PBS(Mean OD±SE)	PPD_AV_(Mean OD±SE)	PPD_BOV_(Mean OD±SE)
**No PTB lesions**	157	3.82	17.20	1.27	0.149±0.010	23.57	28.66	0.128±0.012	0.175±0.015	0.197±0.021
**PTB lesions**	176	22.16	38.64	10.23***	0.358±0.049***	40.34**	15.34	0.144±0.019	0.256±0.025**	0.203±0.020
***Focal***	152	13.16	29.61	1.97	0.170±0.016	36.84	17.11	0.147±0.022	0.235±0.026	0.198±0.021
***Multifocal***	12	58.33	91.67	33.33	0.927±0.308	58.33	0.00	0.088±0.017	0.310±0.129	0.166±0.072
***Diffuse***	12	100.00	100.00	91.67	2.175±0.247	66.67	8.33	0.161±0.037	0.476±0.097	0.302±0.073
***-D.multibacillary***	7	100.00	100.00	100.00	2.440±0.149	85.71	0.00	0.120±0.019	0.546±0.141	0.306±0.105
***-D.intermediate***	4	100.00	100.00	75.00	2.028±0.643	50.00	0.00	0.150±0.060	0.298±0.127	0.196±0.047
***-D.lymphocytic***	1	100.00	100.00	100.00	0.906	0.00	100.00	0.489	0.694	0.700
**Total/Mean**	333	13.51	28.53	6.01		32.43	21.62			

MAP presence was assessed by tissue culture and rtPCR. Paratuberculosis specific antibodies detected by ELISA (AB) and IFN-γ release assay (IGRA) after blank stimulation (PBS) and stimulation with avian (PPD_AV_) and bovine antigens (PPD_BOV_) are shown as mean optical density (OD) values and standard error (SE). Statistical significance (Student t-test): *p<0.05; ** p<0.01; *** p<0.001.

The majority of *focal* lesions appeared in mesenteric LN (88.8%; 135/152) whereas focal granulomas affecting ICV and DI accounted for 2.6% (4/152). In 13 cases *focal* lesions appeared both in LN, ICV and DI tissue sections (8.6%). *Multifocal* lesions were usually observed both in the intestine and in the LN (75.0%; 9/12). *Diffuse* lesions were always found both in the intestinal wall and in the associated LN.

Acid-fast bacilli (AFB) were hardly detected by ZN staining in animals showing *focal* lesions (12.5%, 19/152), if compared to those developing *multifocal* (75.0%; 9/12), or *diffuse* lesions (100.0%; 12/12).

MAP isolation was achieved in 45 cows (13.5%; 45/333) whereas the proportion of MAP specific IS*900* DNA was approximately two times higher (28.5%; 95/333) (Table 3). Of the 157 animals without microscopic lesions, six cows (3.8%; 6/157) had culture-positive tissues and twenty seven (17.2%; 27/157) were positive by rtPCR. Similarly to the distribution of AFB, both microbiological and molecular MAP detection rates greatly increased with the severity of lesions (Table 3).

### Humoral Immune Response, Histopathology and MAP Detection

Twenty animals (6.0%; 20/333) had a positive result in the indirect ELISA (Table 3). Positive results were higher (t-Test; p<0.0001) and more frequent (10.2%; 18/176) (Fisher; p = 0.0004) among animals with PTB lesions than among those without lesions.

As tissue damage increased, seropositivity rate and the antibody levels significantly increased. Only animals with *focal* forms failed to show significant differences in frequency or mean OD readings with the no lesion group. They showed significantly lower antibody means when compared with those of *multifocal* and *diffuse* forms (p<0.0001).

The mean OD of humoral responses among animals with *multifocal* lesions was nearly half of that observed in cows with *diffuse forms* (p<0.0001). Within the three *diffuse* forms considered, the single cow showing *lympho-plasmacytic* enteritis showed a reduced antibody mean compared to *intermediate* (p = 0.0070) and *multibacillary* or *histiocytic* (p<0.0001) forms.

Regarding MAP detection, ELISA positivity was associated with both tissue culture (90.0%; 18/20) and rtPCR (85%; 17/20) positive results (Fisher; p<0.0001) (Table 4).

**Table 4:** Proportion of animals with confirmed MAP isolation or IS*900* DNA detection in tissues according to ELISA and IFN-γ release assay (IGRA) results.

	ELISA		IGRA (PPD_AV_)		IGRA (PPD_BOV_)	
	Neg	Pos	Neg	Pos	Neg	Pos
**%Tissue Culture +**	8.63	90.00	8.89	23.15	15.71	5.56
**% Tissue** **rtPCR +**	24.92	85.00	27.11	31.48	29.50	25.00

### Cell-mediated Immune (CMI) Response, Histopathology and MAP Detection

The specific IFN-γ productions in response to the PPD_AV_ and PPD_BOV_ antigens, as well as the basal IFN-γ (PBS) levels related to González et al. (2005) [24] histopathological lesions previously described are summarized in Table 3. Although increased, no differences in the IFN-γ levels in response to the null-antigen were observed regarding the different types of PTB lesions. On the contrary, the specific IFN-γ levels in response to the avian antigen were increased in the groups with PTB lesions (p_AV_ *= *0.0080; p_AV-PBS_ *= *0.0395). Thirty two per cent (108/333) of the animals showed positive CMI responses against the avian antigen. The frequency of positive animals among the no-lesion ones was 23.6%, but it increased up to 40.3% among animals with any type of PTB lesions (p = 0.0015).

Considering the histopathological form, the highest IFN-γ levels against PPD_AV_ corresponded to *diffuse* lesions (OD value = 0.476±0.097) and particularly, to the single animal classified as a *diffuse lymphocytic* form (OD value = 0.694). The animals with *diffuse* forms had significantly increased specific avian IFN-γ levels in comparison with those without lesions (p_AV_ *= *0.0018; p_AV-PBS_ *= *0.0106) and animals showing *focal* forms (p_AV_ *= *0.0198; p_AV-PBS_ *= *0.0431), but not *multifocal* (p_AV_ *= *0.4577; p_AV-PBS_ *= *0.8599).

With regard to the bovine TB PPD antigen, 72 animals tested positive in the IGRA (21.6%; 72/333); of which only 37.5% had lesions consistent with PTB (27/72). For all these animals, lesions were classified as *focal*, except for one showing *lymphoplasmacytic* enteritis. Similarly, mean IFN-γ productions against PPD_BOV_ increased with tissue damage. In a comparative perspective, higher responses were observed for the PPD_AV_ than to the PPD_BOV_ and no significant differences were detected related to PTB histology.

IGRA-positivity to the avian antigen was associated with an increased proportion of positive-tissue culture animals (Fisher; p = 0.0006) (Table 4). This higher MAP isolation rate was also confirmed for animals testing negative to the bovine antigen (Fisher, p = 0.0307). No differences in the proportion of IS*900* DNA positive results were detected for any of the two antigens (Fisher; p_PPD AV_ = 0.4377; p_PPD BOV_ = 0.5556).

### Relationship between the Specific Antibody and Interferon Gamma (IFN-γ) Production Associated with Histopathology

**Figure 1:**
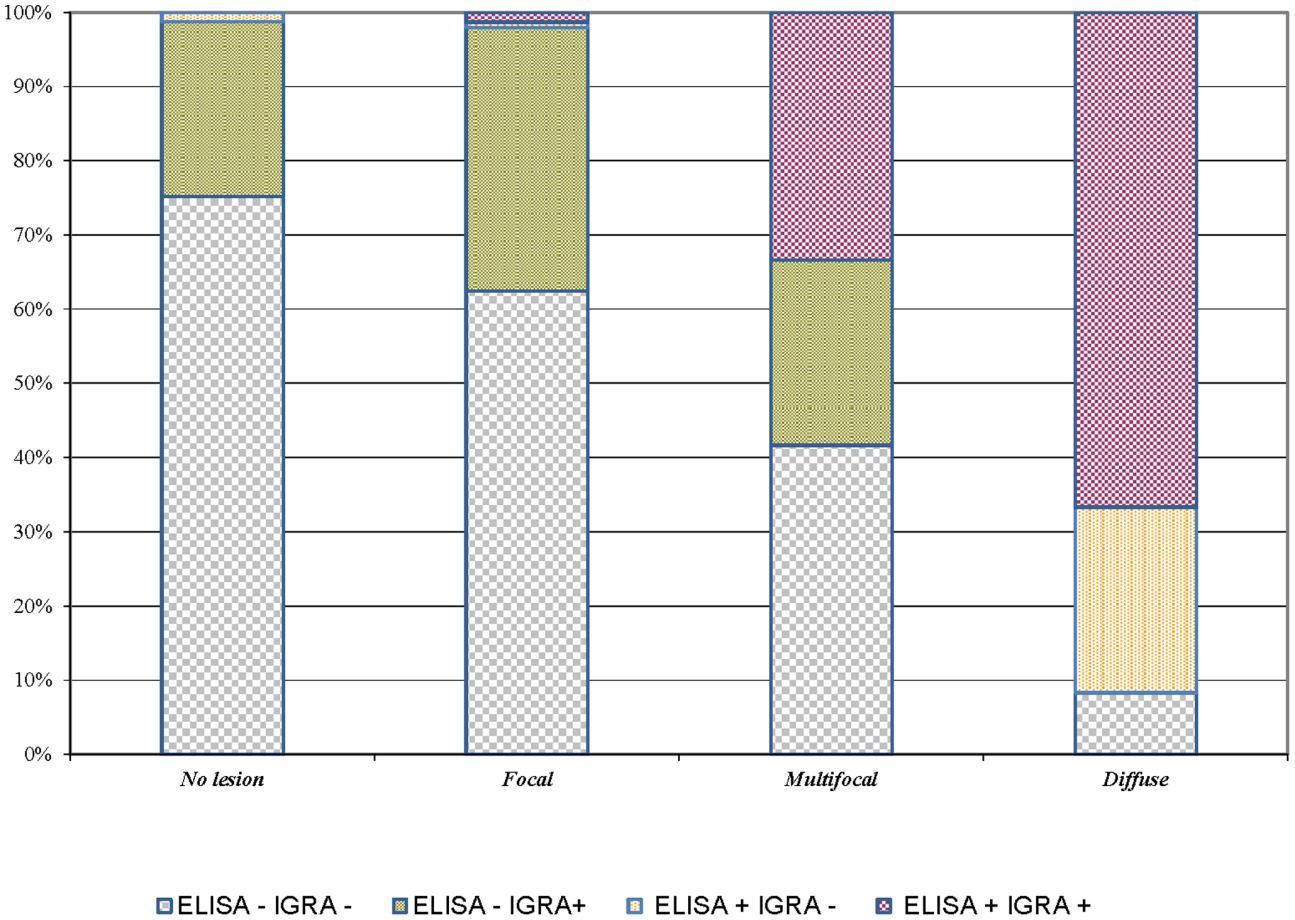
Characterization of immune responses against MAP according to histopathological findings consistent with PTB. Notice how the immunological pattern varies for each pathological form. IGRA: IFN-γ release assay for cell mediated immune response (avian PPD antigen). ELISA: Enzyme-linked immunosorbent assay for humoral immune response (IDEXX Paratuberculosis Screening Ab Test and IDEXX Paratuberculosis Verification Ab Test; IDEXX Laboratories, Inc., Westbrook, ME, USA).

Patterns of immune responses against MAP according to lesion type are shown in Figure 1. Among animals without inflammatory lesions, some type of immune reaction was detected in 24.8% of the animals. Within this group, CMI responses (23.6%; 37/157) predominated over humoral responses (1.3%; 2/157). The presence of PTB lesions was mainly associated with CMI responses (40.3%; 71/176); however, concomitant antibody and IFN-γ productions were detected in 8.0% (14/176) of animals with inflammatory lesions of any type. Conversely, no immune response was detected in 57.4% (101/176) of animals with PTB lesions. The antibody production was closely related to the presence of *diffuse* forms (91.7%; 11/12). In turn, *focal* (35.5%; 54/152) and *multifocal* (25.0%; 3/12) forms were mostly associated with CMI responses. Likewise, the likelihood of detecting combined immune responses (positive to both ELISA and IGRA) was in agreement with the occurrence of advanced tissue damage, while no concomitant antibody and IFN-γ increase was detected among animals without lesions.

Seropositive cows with *multifocal* lesions always had positive responses to the avian antigen. Meanwhile, combined immune response among seropositive cows with *diffuse* lesions accounted for 72.7% (8/11).

### Sensitivity and Specificity of Immunological Tests for Detecting Paratuberculosis Lesions

The sensitivity (Se) and specificity (Sp) estimates of both immunological tests for *focal, multifocal* and *diffuse* PTB forms are shown in Table 5. Both immunological methods lacked in sensitivity for detecting delimited (*focal* and *multifocal)* PTB lesions. However, the ELISA test had a good sensitivity for *diffuse* lesions. Higher estimates of Sp were associated with the ELISA test than with the IGRA that hardly accounted for 77.0%. Agreement between humoral responses and histopathological lesions increased with tissue damage. In fact, both variables had an excellent agreement in the case of *diffuse* lesions (κ = 0.870). Conversely, poor agreements between any form of PTB lesions and the IFN-γ productions were detected.

**Table 5:** Diagnostic efficiency of the ELISA and the IFN-γ release assay (IGRA) for different PTB lesion forms and MAP detection in tissues: Sensitivity (Se) value, Specificity (Sp) value and agreement between tests (Kappa index, κ).

Neg/Pos Reference	Immunological test	% Se	% Sp	Agreement (κ)	Fisher’s exact test (p-value)
**Histopathology**					
No lesion vs *focal*	ELISA	2.0	98.7	0.007 (poor)	0.6805
	IGRA	36.8	76.4	0.134 (poor)	0.0131*
No lesion vs *multifocal*	ELISA	33.3	98.7	0.417 (moderate)	0.0002***
	IGRA	58.3	76.4	0.156 (poor)	0.0144*
No lesion vs *diffuse*	ELISA	91.7	98.7	0.870 (excellent)	<0.0001***
	IGRA	66.7	76.4	0.190 (poor)	0.0031**
**Microbiology in tissues**					
Culture- vs Culture+	ELISA	40.0	99.3	0.513 (moderate)	<0.0001***
	IGRA	55.6	71.2	0.168 (poor)	0.0006***
rtPCR- vs rtPCR+	ELISA	17.9	98.7	0.218 (fair)	<0.0001***
	IGRA	35.8	68.9	0.045 (poor)	0.4377

Interpretation of κ index: 0.00–0.20 poor, 0.21–0.40 fair, 0.41–0.60 moderate, 0.61–0.80 good and 0.81–1.00 excellent agreement. Statistical significance (Fisher’s exact test): *p<0.05; **p<0.01; ***p<0.001.

### Sensitivity and Specificity of Immunological Tests for Detecting MAP

The IGRA test had better sensitivity values for MAP isolation (Se = 55.6%) and IS*900* DNA detection (35.8%) in tissues than the ELISA test, but its specificity was nearly a 30% lower compared with the humoral test (Table 5). Both microbiological methods resulted in poor agreement with the IGRA results, whereas the ELISA test resulted in fair (κ = 0.218) to moderate (κ = 0.513) agreement with the rtPCR and tissue culture results, respectively (Table 5).

## Discussion

Increased specific antibody and IFN-γ levels as well as higher frequencies of positive results were observed among MAP infected animals, in agreement with previous reports [30]. This overall enhanced immunological response rate associated with PTB lesions was mostly due to the responses in the IGRA (40.3%), since only 10.2% of the animals reacted in the indirect ELISA. However, nearly half of animals with characteristic histopathological lesions did not react in any of these assays (57.4%). Although some degree of failure in the immunological detection of animals showing PTB lesions has been described [31], [32], our current estimate is worse. This could be likely associated with the different frequencies of types of PTB lesions in each study, and especially the high frequency of *focal* types in the current study.

In apparently PTB-free animals, immune reactivity against MAP basically consisted in CMI responses with few individuals testing positive in the ELISA (1.3%). Although the prevalence of MAP in these histologically normal individuals was estimated between 3.8% (culture) and 17.2% (rtPCR), it is noteworthy that MAP was only confirmed in three of the 37 animals showing CMI profiles (8.1%) and in none of the ELISA-positive ones (data non shown). Interestingly, animals showing *focal* lesions presented similar immune responses. Also, the positivity to the IGRA increased the likelihood of detecting MAP in tissues if compared with the humoral test. This suggests that MAP exposure or infection would lead to an increased IFN-γ release which would thus in most cases restrict MAP multiplication keeping the animals either as apparently free of PTB or with minimal *focal* granulomatous changes. On the other hand, *multifocal and diffuse* forms strongly differed from apparently free and *focal* cases in the humoral response (p<0.0001) but not so much in the cell-mediated one (Table 3; Figure 1).

Humoral data from this study are not unexpected since it is well known that antibody ELISA tests have a very low sensitivity for subclinically infected animals [33]–[35]. In turn, the overall better sensitivity of the IGRA, which was able to detect about one third of the *focal* forms but also near two thirds of the more advanced forms, was less predictable according to the classical humoral/cellular balance paradigm. In this study, avian positive results were significantly more frequent among individuals with advanced enteritis than among those showing delimited lesions (p = 0.0243). The same atypical tendency has been found after MAP sonicate-stimulation of peripheral blood mononuclear cell (PBMC) and mesenteric lymph node (MLN) cultures of cull cows from MAP infected herds which showed severe lesions [36]. Not only histological findings but also the high rate of reactions to bovine PPD suggested that the CMI response was rather unspecific and therefore, of low diagnostic utility. In fact, and in agreement with the findings of Alvarez et al. (2009) [37] reporting a decrease of sensitivity of cellular immunity based tests for TB diagnosis, clear TB positive results were obtained up to 28.7% of apparently free, 17.1% of *focal* forms and less of the other forms, except for the *lymphocytic* or *lymphoplasmacytic* ones. This reduced rate of positive results in the comparative interpretation of advanced forms indicates that it could be the calculation artifact caused by the increased avian reference values, rather than a true interference in the immune response; what might cause the reported loss of sensitivity of IGRA in PTB infected herds [37]. Anyway, even following the test manufacturer instructions for the use of a 0.1 cut-off, the use of the IGRA alone would have resulted in falsely scoring 19.1% of healthy animals and 12.5% of cows with *focal* forms (overall 15.0%) as TB positive. Albeit no specific investigation of the TB status of the animals was carried out, it is highly unlikely that any of them could have had such an infection since Spanish cattle is subjected to yearly TB official eradication campaigns and no documentary nor pathological evidence of TB was recorded for any of the animals included in the present study. This further supports the low specificity of the IGRA and the correctness of restricting its use to TB-confirmed herds as well as not recommending it for general TB screening [37], [38]. Moreover, false positive reactions to PPD_BOV_ have been often related to PTB vaccinated animals [38]–[40]; however, no interference with animals sensitive to PPD_BOV_ should be expected in this study because although PTB vaccination in small ruminants is allowed under veterinary control, it is explicitly prohibited for cattle by the Spanish Animal Health authorities.

Immunological dynamics observed in this study would fit a recently proposed pathogenesis model of MAP infections defined by two categories: *latent* (*focal*) and *patent* (*multifocal* and *diffuse* forms) [41]. In that context, *focal* forms would represent a condition of certain natural resistance or premunition [32] sustained by the presence of a continuous confined inflammatory focus, while the low proportion of *multifocal* forms (6.8% of all forms) would rather represent a transient state in the tapering trend towards each of the progressively rarer *diffuse* final patent forms. However, our findings do not support the standard model where IFN-γ release would be predominant in early stages of disease or increased resistance to it and decreased in more advanced forms, where humoral responses would be the hallmark [6], [8], [11]. In fact, our data showed a mixed Th1/Th2 response that simultaneously increased as the extension and severity of lesions grew, a finding that is also supported by other recent observations [5], [15]. Although, the low number of the three *diffuse* lesion types make it difficult to draw further conclusions on their meaning, it could be postulated that they represent deviations from the main pattern, due to slight differences in the inflammatory pathways or in the degree of previous exposure to MAP or other mycobacteria. The only case of *lymphocytic* PTB where both IFN-γ and antibody responses were highly increased, results particularly interesting because it was positive to bovine TB rather than to PTB in a comparative interpretation. More strikingly, there was a nearly fourfold increase of basal IFN-γ levels in the non-stimulated plasma of this animal. Therefore, this feature suggests that either there is a much increased release of IFN-γ from the inflamed tissues, which would be compatible with the *lymphoplasmacytic* character of the infiltrate; or it has some degree of impairment of IFN-γ catabolism, which would be compatible with a model of pathogenesis already suggested for human Crohn’s disease (CD), related to lack or diminished functionality of IFN receptors [42], [43]. Additionally, apart from being the morphologically closest form to that of human CD, in another study in parallel to the one presented here, this immunopathological form showed a surprisingly narrow range of variability in the age at slaughter, suggestive of a nearly unimpaired course of the disease [3]. Hence, unsuccessful but strong cell-mediated immune responses occurring in *lymphoplasmacytic* lesions [24], [44], [45] might represent the purest trans-specific form of intestinal inflammatory disease in ruminants, similar to those observed in monogastric domestic species [46], [47] and humans [48]. In this sense, we think that replacing the *lymphocyti*c and *multibacillary* terms proposed by González et al., (2005) [24] with the denomination of *lymphoplasmacytic* and *hystiocytic*, respectively by using exclusively pathological terms would be more descriptive and scientifically useful and would also unify terminology with inflammatory bowel disease (IBD), throughout all species presenting this type of disease independently of the presence of mycobacteria.

Contrary to Brady et al. (2008) [49] findings, we did not confirm the presence of MAP in all affected tissues. In fact, MAP was detected in the 32.9% of the *focal* cases when combining both microbiological tests which was similar to the rates reported for tissue culture and PCR by González et al. (2005) in this type of lesions [24]. It is possible that in the study reported by Brady et al. (2008) [49] the likelihood of identifying MAP could have been increased because of two reasons: the high prevalence of clinically infected animals and the multisampling of tissues which were submitted to histopathological and culture procedures. Furthermore, it has been postulated that some bacterial clearance or reduction could occur among MAP infected cattle modulated by IL-10 cytokine levels [50].

In terms of practical diagnostic use, the combination of both blood tests did not result in an increased efficiency (Figure 1). Taken separately (Tables 3 and 5) and referred to histopathology, the ELISA test showed a good specificity but a poor sensitivity if delimited forms are taken into account. The test was particularly inefficient for *focal* forms that were the vast majority of cases (86.4%). However, it detected one third of *multifocal* forms and was very efficient for *diffuse* forms (Se = 91.7%; excellent agreement: κ = 0.870), of which only one *intermediate* case was missed. Moreover, despite the overall lack of sensitivity for detecting MAP infected tissues, the ELISA showed moderate agreement with the bacteriological culture (κ = 0.513) and the seropositivity was strongly associated with both MAP isolation (90%) and IS*900* amplification (85%). The IGRA was better at detecting individuals with delimited forms (Se*_focal_* = 36.8%; Se*_multifocal_* = 58.3%) as well at identifying those with viable bacterial loads in tissues (Se = 55.6%) than ELISA. However, since it always was related to low specificity values (68.9% to 76.4%) and showed poor agreement with the reference methods results (microbiology and histopathology), the IGRA appears not to be an adequate tool to identify MAP infected, infectious or affected individuals although it might be useful to evaluate the level of MAP exposure in young animals (12–24 months), which is in agreement with Jungersen et al. (2012) [51] suggestions.

From an infection control perspective, most animals testing positive to the IGRA had no serious tissue damage (86.1%) nor harbored MAP (63.9%). In turn, in 75.0% of seropositive animals *patent* forms of infection were observed and MAP isolates were confirmed in 90% of ELISA-positive cases. According to these results, the contagiousness would be particularly high in those showing Th2 or combined Th1/Th2 responses against MAP.

In summary, our results indicate that in Friesian cattle most MAP infections present as *latent* forms (86.4%) corresponding to the *focal* granulomatous lesions type defined by González et al. (2005) [24] and linked to limited specific immunological responses against MAP. Only a few animals developed advanced lesions associated with a humoral response and *patent* disease. Although the development of PTB lesions was consistent with the traditional dynamics of antibody production and increased IFN-γ levels described for bovine PTB, the polarization of CMI response did not appear to be so clear in our cases. Finally, our results confirm the unreliability of the IGRA test for PTB diagnostic because its low specificity and support restricting its use to specific circumstances in bovine TB schedules.
